# Laparoscopic one-stage vessel-sparing orchiopexy and two-stage laparoscopic Fowler-Stephens orchiopexy: a comparative study

**DOI:** 10.3389/fped.2026.1781219

**Published:** 2026-04-17

**Authors:** Thuy Nguyen Thi Mai, Khang Do Van

**Affiliations:** 1 Department of Urology, Vietnam National Children's Hospital, Hanoi, Vietnam; 2 Department of Surgery, Hanoi Medical University, Hanoi, Vietnam

**Keywords:** Fowler-Stephens technique, intra-abdominal undescended testis, laparoscopy, orchiopexy, vessel-sparing orchiopexy

## Abstract

**Objective:**

To compare the surgical outcomes of laparoscopic one-stage vessel-sparing orchiopexy (1S-LSO) and two-stage Fowler-Stephens laparoscopic orchiopexy (2S-LFSO) in the management of intra-abdominal undescended testes (IAUT) located less than 2.5 cm from the deep inguinal ring.

**Materials and methods:**

A retrospective study was conducted on 75 intra-abdominal undescended testes (IAUT) in 65 pediatric patients who underwent laparoscopic orchiopexy between January 2021 and June 2024. Data collected included patient age, operative duration, perioperative complications, postoperative testicular position, and testicular volume. Outcomes were compared between the 1S-LSO group and 2S-LFSO group.

**Results:**

The median age at surgery was 33 months (interquartile range: 17–74 months). 45 testes were managed with 1S-LSO and 30 with 2S-LFSO, with no intraoperative or early postoperative complications observed. The follow-up rate was 77.3% (58/75 testes), with a mean follow-up duration of 18.9 ± 7.1 months. The rate of successful scrotal placement was high in both groups: 78.8% for 1S-LSO and 88.0% for 2S-LFSO (*p* = 0.32). Similarly, the rate of testicular atrophy was not significantly different: 3.0% for 1S-LSO and 8.0% for 2S-LFSO (*p* = 0.57). The 1S-LSO group demonstrated a statistically significant increase in postoperative testicular volume (preoperative median volume 0.34 mL vs. postoperative volume 0.38 mL, *p* = 0.022), whereas no significant within-group change was observed in the 2S-LFSO group (0.35 mL vs. 0.33 mL, *p* = 0.653).

**Conclusions:**

Both 1S-LSO and 2S-LFSO are safe and highly effective for the treatment of IAUT. Although success and atrophy rates did not differ significantly between groups, the significant increase in postoperative testicular volume observed only in the 1S-LSO group suggests a possible advantage in postoperative testicular growth during the observed follow-up period. The choice of technique should be guided by careful anatomical and intraoperative assessment, favoring the single-stage vessel-sparing procedure when conditions permit.

## Introduction

Cryptorchidism (undescended testis) is among the most common congenital anomalies in male infants, with an incidence ranging from 1% to 4.6% in full-term newborns (1). If not surgically corrected in a timely manner, this condition may lead to serious consequences, including testicular torsion, cancer, impaired fertility, and psychological distress during adolescence (2, 3).

The surgical history of cryptorchidism dates back to 1877, when Thomas Annandale performed the first successful orchiopexy. Subsequent refinements by surgeons such as Max Schüller and Arthur Dean Bevan established open inguinal orchiopexy as the standard treatment for palpable undescended testes located in the inguinal canal. However, for nonpalpable intra-abdominal testes, the open approach presented significant limitations: it required an extensive abdominal incision and often proved unnecessary in cases where the testis was already atrophic. In 1976, N. Cortesi and colleagues were the first to describe the use of laparoscopy to successfully diagnose bilateral intra-abdominal undescended testes (IAUT) (4). Following Cortesi's report, laparoscopy served as the primary diagnostic tool for more than a decade. The transition from diagnosis to therapeutic application was marked by David A. Bloom in 1991, who described the laparoscopic technique for performing the first stage of the two-stage Fowler-Stephens orchiopexy, a procedure originally introduced via open surgery in 1959 (5, 6). Only a year later, Jordan and Winslow published the first series of complete single-stage laparoscopic orchiopexies. Since then, laparoscopy has gradually become the gold standard for both diagnosis and treatment of IAUT in most pediatric surgical centers worldwide. Nevertheless, the choice between a one-stage or two-stage laparoscopic approach, and how this decision influences surgical and functional outcomes, remains a topic of ongoing debate. To address this issue, we conducted a retrospective study of pediatric patients with IAUT who underwent laparoscopic orchiopexy at a high-volume pediatric urology department between January 2021 and June 2024.

## Materials and methods

### Study population

This study included Vietnamese pediatric patients diagnosed with IAUT who underwent laparoscopic orchiopexy between January 1, 2021, and June 30, 2024.

### Inclusion criteria

- Male patients under 16 years of age (at the time of surgery) diagnosed with IAUT and treated by laparoscopic orchiopexy in the study period at a high-volume pediatric urology department in Hanoi, Vietnam.
- Intraoperative findings confirmed that the affected testis was located within 2.5 cm of the deep inguinal ring.

### Exclusion criteria

- Patients who had previously undergone surgical treatment for undescended testis at other medical facilities.
- Patients with known congenital syndromes or disorders affecting gonadal development, such as Prader-Willi syndrome, Klinefelter syndrome, persistent Müllerian duct syndrome, or other disorders of sexual differentiation.

### Surgical technique

All procedures were performed under general anesthesia by senior pediatric urologists (with more than 10 years of experience). A 5 mm trocar was placed at the umbilicus for the camera operator, and two additional 3 mm trocars were placed at the mid-clavicular line for working instruments. Upon laparoscopy, the testis, its gross appearance and its vasculature were assessed by the surgeon. A tape ruler was inserted into the abdomen to measure the distance between the testis and the deep inguinal ring. An intraoperative traction test was performed, by gently grasping and pulling the testis toward the contralateral deep inguinal ring. If the testis could reach the contralateral inguinal ring without tension, 1S-LSO was indicated. If not, 2S-LFSO was selected. The traction test was performed immediately after diagnostic laparoscopy and measurement of the distance from the testis to the deep inguinal ring, but before division of the gubernaculum, extensive retroperitoneal mobilization, or clipping/division of the spermatic vessels. This sequence was intentionally used to assess native cord length and to preserve the feasibility of a staged Fowler-Stephens procedure when the testis could not be advanced without undue tension.

#### Single-stage vessel-sparing laparoscopic orchiopexy

The procedure began with the division of the gubernaculum and lateral peritoneal attachments. Extensive retroperitoneal mobilization was performed, carefully dissecting the spermatic vessels proximally to the lower pole of the kidney to maximize cord mobility while preserving the “spermatic triangle” to protect the vascular integrity.

#### Two-stage laparoscopic Fowler-Stephens orchiopexy

In the first stage, the spermatic vessels were identified, clipped, and divided high cranially, well above the testis, to allow for the development of collateral circulation. The second stage would be performed approximately 6 months after the first stage. A wide triangular peritoneal flap, encompassing the testis and its collateral blood supply (based on the deferential and cremasteric arteries), was created and mobilized to bridge the gap to the scrotum.

In both techniques, a new pathway was created through the inguinal canal (or medial to the inferior epigastric vessels, if required for the Fowler-Stephens approach). The mobilized testis was grasped and guided into the scrotum without torsion. Finally, the testis was placed in a sub-dartos pouch and fixed with non-absorbable sutures to prevent retraction.

### Study design and methods

This was a retrospective comparative study of a longitudinal case series. All patients within the study period, including all eligible testes managed by either laparoscopic one-stage vessel-sparing orchiopexy (1S-LSO) or two-stage Fowler-Stephens laparoscopic orchiopexy (2S-LFSO), were recruited into the study. As this was a retrospective, non-randomized study, all consecutive eligible patients during the study period were included to reduce investigator-driven case selection. The choice between 1S-LSO and 2S-LFSO was made intraoperatively according to anatomic feasibility, including testicular position, native spermatic vessel length and mobility, and the result of the traction test. No age-matching or propensity score matching was performed, because treatment allocation was determined by intraoperative anatomy rather than by preoperative random assignment. Accordingly, the comparative analyses were intended to reflect real-world surgical decision-making and should not be interpreted as evidence of causality.

Clinical data were collected from medical records and postoperative follow-up visits. The following parameters were recorded: patient age, laterality of undescended testis, intraoperative testicular position, type of procedure (1S-LSO vs. 2S-LFSO), operative time (minutes), intraoperative and early postoperative complications (such as bleeding or trocar-site infection), and length of hospital stay (days). Postoperative follow-up was conducted at least six months after surgery to assess testicular position and size by ultrasonography, as well as later complications, including inguinal hernia, trocar-site hernia, and postoperative intestinal obstruction.

Testicular volume was calculated using the modified Lambert formula: V = L × W² × 0.71 (mL), where L represents testicular length (cm) and W represents width (cm). This formula is based on the rotational ellipsoid model proposed by Prader and later referenced by Sotos and Tokar (7). Lambert formula provides the most accurate estimation of true testicular volume in pediatric populations, with low inter-observer variability (8). Therefore, this method was specifically selected to minimize measurement variability and enhance sensitivity when evaluating the inherently small volume changes characteristic of prepubertal undescended testes. Postoperative testicular size was assessed by scrotal ultrasonography at follow-up visits conducted at least 6 months after definitive orchiopexy. For patients undergoing 2S-LFSO, the postoperative interval was calculated from the second-stage orchiopexy.

All data were encoded, entered, and analyzed using SPSS version 26.0 statistical software package (IBM Corp., Armonk, NY, USA). Descriptive variables were described as mean ± standard deviation (SD), median (interquartile range—IQR) and percentages. Categorical variables were compared with Fisher's exact test, comparison of continuous variables were done with Mann–Whitney *U* test, and comparison between pre-operative and post-operative values were done with Wilcoxon signed-rank test. A *p*-value less than 0.05 (*p* < 0.05) was considered statistically significant with a confidence interval of 95%.

### Ethical considerations

All patient information was anonymized and kept confidential throughout the study. No personal identifiers were disclosed in any form. Ethical approval for this study was obtained from Vietnam National Children's Hospital Institutional Review Board (IRB-VN01037/IRB00011976/FWA00028418). Written informed consent was obtained from legal guardians of the patients prior to data collection.

## Results

During the study period, 65 patients with a total of 75 undescended testes were included. Of these, 55 patients (84.6%) had unilateral, and 10 patients (15.4%) had bilateral intra-abdominal undescended testes. The median age at surgery was 33 months (IQR: 17–74 months). Group-specific ages are presented in Table 1 with values of 29 (15–70) months for 1S-LSO and 33 (23–82) months for 2S-LFSO. Nineteen patients (29.2%) underwent early surgery between 6 and 18 months of age.

**Table 1:** Intraoperative findings and early postoperative outcomes.

Parameter	1S-LSO	2S-LFSO
Number of patients, *n*	38	27
Number of testes, *n*	45	30
Median age at surgery (IQR), months	29 (15–70)	33 (23–82)
Status of the deep inguinal ring, *n* (%)		
Patent	37 (82.2)	22 (73.3)
Closed	1 (2.2)	5 (16.7)
Not described	7 (15.6)	3 (10.0)
Mean operative time for unilateral procedure (X¯ ± SD, minutes)		
Stage I	42.2 ± 10.4	25.3 ± 4.1
Stage II	–	39.7 ± 9.3
Intraoperative complications, *n* (%)	0 (0)	0 (0)
Early postoperative complications, *n* (%)	0 (0)	0 (0)

All 75 affected testes in the study were located within 2.5 cm of the deep inguinal ring. Among these, 45 testes underwent single-stage laparoscopic vessel-sparing orchiopexy (1S-LSO), while 30 testes were treated using the two-stage Fowler-Stephens laparoscopic technique (2S-LFSO), with an interval of approximately six months between stages (Table 1).

The overall follow-up rate was 77.3% (58/75 testes) with a mean follow-up duration of 18.9 ± 7.1 months. Among these, postoperative ultrasonographic volume measurements were available in 55 testes, and paired preoperative-postoperative volume data were available in 50 testes. The comparative postoperative outcomes regarding testicular position, volume, and presence of atrophy between the two surgical techniques are presented in Table 2. Testicular atrophy was defined as a ≥50% reduction in volume compared with preoperative measurement or when the affected testis volume was <25% of the contralateral healthy testis (9). 2S-LFSO achieved a higher rate of successful scrotal positioning compared with the single-stage vessel-sparing procedure. Although the postoperative testicular volume tended to be smaller in the two-stage group, but this was not statistical significant (*p* > 0.05).

**Table 2:** Comparison of postoperative follow-up outcomes between the two surgical groups.

Parameter	1S-LSO	2S-LFSO	*p*
Number of testes, *n* (missing)	33 (3)	25 (0)	–
Follow-up duration, mean ± SD, months	20.7 ± 7.2	16.8 ± 6.3	–
Located in the scrotum, *n* (%)	26 (78.8)	22 (88.0)	0.32^a^
High position, *n* (%)	6 (18.2)	1 (4.0)	0.13^a^
Atrophy, *n* (%)	1 (3.0)	2 (8.0)	0.57^a^
Postoperative testicular volume, median (IQR), mL	0.38 (0.27–0.55)	0.33 (0.26–0.46)	0.35^b^

*Missing*: Testes without postoperative ultrasound volume measurement were excluded from the analysis.

a Fisher's exact test.

^b^Mann–Whitney *U* test.

Among testes with postoperative follow-up, ultrasonographic reassessment was performed after a mean follow-up of 20.7 ± 7.2 months in the 1S-LSO group and 16.8 ± 6.3 months in the 2S-LFSO group. No statistically significant difference was observed between preoperative and postoperative testicular volumes in the two-stage Fowler-Stephens group (*p* > 0.05). In contrast, 1S-LSO group demonstrated a slight but statistically significant increase in postoperative testicular volume (*p* < 0.05) (Table 3).

**Table 3:** Comparison of preoperative and postoperative testicular volume by surgical technique.

Surgical method	*n* (missing)	Preoperative volume [Median (IQR), mL]	Postoperative volume [Median (IQR), mL]	*p* ^a^
1S-LSO	33 (6)	0.34 (0.25–0.45)	0.38 (0.27–0.55)	0.022
2S-LFSO	25 (2)	0.35 (0.28–0.49)	0.33 (0.26–0.46)	0.653
Total	58 (8)	0.34 (0.26–0.43)	0.36 (0.27–0.52)	0.236

*Missing:* Testes with incomplete volume data (missing either preoperative or postoperative measurement) were excluded from analysis.

^a^Wilcoxon signed-rank test.

No cases of late postoperative complications such as inguinal hernia, trocar-site hernia, or adhesive intestinal obstruction were recorded.

## Discussion

In laparoscopic orchiopexy for IAUT, one of the most critical intraoperative decisions is whether to proceed with a single-stage or two-stage intervention. The distance between the testis and the deep inguinal ring has been widely regarded as a key predictor of successful testicular descent. However, the international literature has yet to reach a clear consensus regarding the precise cutoff value for this distance. Reported thresholds vary among authors, with Wang et al. suggesting that single-stage orchiopexy is feasible when the distance is less than 2.5 cm (10). Elsherbeny et al. proposed a 2 cm limit (11). Meanwhile, He et al. recommended a more conservative threshold of 1 cm (12). These variations indicate that the distance to the deep inguinal ring should not be considered the sole determinant in selecting the operative approach. Instead, it must be evaluated in conjunction with other intraoperative and anatomical factors, such as testicular mobility, vascular length, and the presence of collateral blood supply. The present study focused on cases in which the intra-abdominal testis was located within 2.5 cm of the deep inguinal ring. Even within this seemingly favorable range, our findings emphasize that the optimal surgical approach should be individualized, considering the comprehensive anatomical and technical context of each patient.

Furthermore, beyond testicular position, the tension test has been widely used by many authors to determine the feasibility of single vs. two-stage laparoscopic orchiopexy. Pogorelić et al. emphasized that the fundamental prerequisite for a single-stage procedure is the ability to mobilize the testis across to the contralateral deep inguinal ring without tension on the spermatic vessels (13). Similarly, He et al. confirmed that this maneuver serves as a valuable predictor for the feasibility of single-stage orchiopexy (12). However, in both studies, this maneuver was performed after extensive dissection and high mobilization of the testicular vessels. This raises an important consideration: if the testis cannot be advanced to the contralateral deep inguinal ring without tension, what should be the optimal alternative? He et al. pointed out that, in practice, this assessment often occurs only after the vessels have already been widely dissected at which point the option of performing a two-stage Fowler-Stephens procedure may no longer be viable (12). Therefore, he proposed using the distance between the testis and the deep inguinal ring as a more objective pre-dissection indicator for selecting the operative strategy. On the other hand, several authors have advocated switching to the Shehata technique instead of Fowler-Stephens under these circumstances (14). The rationale is that traction testing and wide dissection may already compromise the vascular integrity of the spermatic cord, and further division of the testicular vessels could increase the risk of ischemia and atrophy. Additionally, the potential for postoperative adhesions during the second-stage operation should not be overlooked. Lin et al. suggested that, in such cases, the Shehata technique provides a safer alternative because it avoids additional manipulation of the previously dissected vessels (15). Similarly, Pogorelić et al. did not employ the Fowler-Stephens approach in patients with a short spermatic cord but instead recommended Shehata's traction technique when sufficient length could not be achieved for a single-stage repair (13). Unlike protocols in which the traction test is performed only after extensive vessel dissection, our traction test was applied before wide mobilization of the spermatic vessels. We adopted this sequence to assess native cord length while preserving the option of proceeding to 2S-LFSO when the testis could not be advanced without undue tension.

In the present study, among 75 intra-abdominal testes located within 2.5 cm of the deep inguinal ring, 45 underwent 1S-LSO and 30 underwent the two-stage procedure. This distribution reflects real-world surgical practice, in which the optimal approach for low intra-abdominal testes remains a matter of ongoing debate. The decision is influenced not only by anatomical factors such as testicular position and vascular length but also by the surgeon's experience, preference, and technical proficiency. A global survey conducted by Shehata et al. among 436 members of the World Federation of Associations of Pediatric Surgeons (WOFAPS) clearly demonstrated this diversity in surgical opinion (16). For low intra-abdominal testes (defined by Shehata as within 3 cm of the deep inguinal ring), the proportions of surgeons favoring the two-stage Fowler-Stephens approach (36.0%) and those performing single-stage vessel-sparing orchiopexy (35.1%) were nearly identical, while 18.4% preferred Shehata technique. These figures underscore that even when anatomical criteria appear relatively well defined, the ultimate choice of surgical method remains strongly influenced by each surgeon's familiarity and confidence with their preferred technique.

We observed that the choice of surgical technique should be carefully balanced between the goal of achieving successful scrotal placement and the risk of damaging the testicular parenchyma. Regarding the ability to achieve the desired position, the two-stage Fowler-Stephens laparoscopic orchiopexy produced more favorable results, with 88.0% of testes located in the scrotum compared with 78.8% in the single-stage vessel-sparing group. Only one case (4.0%) of high-positioned testis was recorded in the 2S-LFSO group, whereas this rate was 18.2% in the 1S-LSO group. Although the difference was not statistically significant (*p* = 0.13), the trend suggests that 2S-LFSO may achieve a better rate of testicular descent, particularly in cases with a short spermatic cord. However, this positional advantage appeared to be associated with a tendency toward smaller postoperative testicular volume. The atrophy rate in the 2S-LFSO group was 8.0%, higher than the 3.0% observed in the 1S-LSO group. The median postoperative testicular volume in the 2S-LFSO group (0.33 mL) was also lower than that in the 1S-LSO group (0.38 mL), although there was no statistically significant difference in both atrophy rate (*p* = 0.57) and volume (*p* = 0.35).

A single-center retrospective study by Alam et al. conducted at Nicklaus Children's Hospital (USA) compared 50 cases of single-stage laparoscopic orchiopexy and 35 cases of two-stage Fowler-Stephens procedures. The authors reported a statistically significant difference in postoperative testicular position, with the Fowler-Stephens group achieving superior results: no cases of high-positioned testes compared with 10 cases (20.0%) in the 1S-LSO group (OR = 0.05) (17). Our findings showed a similar trend, with the 2S-LFSO group demonstrating a markedly lower rate of high-positioned testes (4.0%) compared with the 1S-LSO group (18.2%). Although there was no statistical significance in this difference in our series (*p* = 0.13), this may be attributed to a smaller follow-up sample size (*n* = 58 vs. *n* = 85 in Alam's study). Notably, our 18.2% rate of high-positioned testes closely mirrors Alam's 20.0%. Regarding testicular atrophy, Alam et al. found no statistically significant difference between the two techniques, with rates of 11.4% (4/35) in the 2S-LFSO group and 8.0% (4/50) in the 1S-LSO group (OR = 1.48). These results are consistent with our study. The overall atrophy rate in our cohort was lower, particularly in the 1S-LSO group (3.0% vs. 8.0% in Alam's series) (17). This discrepancy may be explained by differences in patient selection: our study included only testes located within 2.5 cm of the deep inguinal ring, whereas Alam's series also included higher-positioned intra-abdominal testes, which are known to carry a greater risk of postoperative atrophy.

However, our study contributes an important aspect not addressed by Alam et al. the change in testicular volume over time. We observed a statistically significant increase in postoperative testicular volume in the 1S-LSO group compared with preoperative measurements (*p* = 0.022), whereas the 2S-LFSO group showed virtually no change (*p* = 0.653). This finding suggests that although the rates of testicular atrophy did not differ between the two techniques, preservation of the native spermatic vessels in single-stage vessel-sparing orchiopexy may facilitate better postoperative catch-up growth of the testis. In contrast, the Fowler-Stephens technique, which relies on collateral circulation from the deferential and cremasteric arteries, may provide sufficient perfusion to maintain testicular viability and prevent atrophy but may not deliver optimal vascular flow to promote substantial growth during the follow-up period. The testicular volumes of the 1S-LSO group were also larger than of the 2S-LFSO group (0.38 vs. 0.33, *p* = 0.35). Although this between-group difference was not statistically significant, the observed pattern may suggest less postoperative catch-up growth after vessel division than after vessel preservation; however, this interpretation remains exploratory. Based on these results, for low intra-abdominal testes with adequate vessel length to reach the scrotum without tension, single-stage vessel-sparing orchiopexy should be the preferred approach, as it preserves physiological perfusion and minimizes the risk of postoperative atrophy. Conversely, the two-stage Fowler-Stephens procedure should be reserved for cases with a short spermatic cord. Applying 2S-LFSO in anatomically favorable situations for single-stage descent may be unnecessary. Nevertheless, this volumetric finding should be interpreted cautiously. In prepubertal children, absolute testicular volume is small, and ultrasonographic estimates may be influenced by measurement variability and the calculation formula used. Therefore, the increase observed in the 1S-LSO group should be considered a supportive indicator of postoperative growth during follow-up rather than definitive evidence of superior long-term endocrine or fertility outcomes.

## Limitations

This study has several limitations. First, the retrospective, single-center design is susceptible to selection bias and confounding by indication, because the choice of operative technique was based on intraoperative anatomy rather than randomized allocation. Consequently, testes treated with 2S-LFSO may have represented more technically challenging cases. Second, no matching or propensity score adjustment was performed, which limits the strength of direct between-group inference. Third, follow-up was incomplete for a proportion of testes, and testicular volume measurements were not available for all cases. Finally, the unit of analysis was the testis rather than the patient, and some patients contributed bilateral testes. Larger prospective studies with standardized postoperative ultrasonographic assessment are needed to confirm these findings.

## Conclusion

Both single-stage vessel-sparing laparoscopic orchiopexy and two-stage Fowler-Stephens laparoscopic orchiopexy are safe and effective techniques for the management of intra-abdominal undescended testes. The decision between a single-stage and a two-stage procedure should be based on a comprehensive assessment of multiple factors, including anatomical characteristics and intraoperative findings. When anatomical conditions are favorable, the single-stage vessel-sparing approach should be prioritized.

## Data Availability

The raw data supporting the conclusions of this article will be made available by the authors, without undue reservation.
